# Expression and purification of the receptor‐binding domain of SARS‐CoV‐2 spike protein in mammalian cells for immunological assays

**DOI:** 10.1002/2211-5463.13754

**Published:** 2024-01-24

**Authors:** Edit Ábrahám, Csaba Bajusz, Annamária Marton, Attila Borics, Thandiswa Mdluli, Norbert Pardi, Zoltán Lipinszki

**Affiliations:** ^1^ MTA SZBK Lendület Laboratory of Cell Cycle Regulation, Institute of Biochemistry HUN‐REN Biological Research Centre Szeged Hungary; ^2^ National Laboratory for Biotechnology, Institute of Genetics HUN‐REN Biological Research Centre Szeged Hungary; ^3^ Laboratory of Chemical Biology, Institute of Biochemistry HUN‐REN Biological Research Centre Szeged Hungary; ^4^ Department of Microbiology University of Pennsylvania Philadelphia PA USA

**Keywords:** ELISA, Expi293F mammalian cells, mRNA‐LNP vaccination, protein purification, recombinant RBD, SARS‐CoV‐2 spike

## Abstract

The receptor‐binding domain (RBD) of the spike glycoprotein of SARS‐CoV‐2 virus mediates the interaction with the host cell and is required for virus internalization. It is, therefore, the primary target of neutralizing antibodies. The receptor‐binding domain soon became the major target for COVID‐19 research and the development of diagnostic tools and new‐generation vaccines. Here, we provide a detailed protocol for high‐yield expression and one‐step affinity purification of recombinant RBD from transiently transfected Expi293F cells. Expi293F mammalian cells can be grown to extremely high densities in a specially formulated serum‐free medium in suspension cultures, which makes them an excellent tool for secreted protein production. The highly purified RBD is glycosylated, structurally intact, and forms homomeric complexes. With this quick and easy method, we are able to produce large quantities of RBD (80 mg·L^−1^ culture) that we have successfully used in immunological assays to examine antibody titers and seroconversion after mRNA‐based vaccination of mice.

## Graphical abstract

This research protocol provides a step‐by‐step guide for expressing and purifying the receptor‐binding domain (RBD) of the SARC‐CoV‐2 spike protein. The receptor‐binding domain is expressed in mammalian cell culture, purified by one‐step affinity chromatography (IMAC), and then concentrated and subjected to quality control. The intact protein can be used in immunological assays (ELISA) to test different antibody‐containing samples.

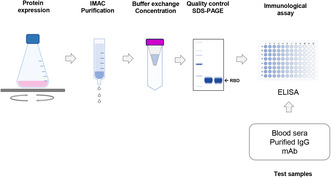

**Abbreviations**
aa: amino acid
AD: alcohol dehydrogenase
BSA: bovine serum albumin
CA: carbonic anhydrase
CD: circular dichroism
COVID‐19: Coronavirus disease 2019
DNA: deoxyribonucleic acid
*E. coli*
: *Escherichia coli*
ELISA: enzyme‐linked immunosorbent assay
ELISpot: enzyme‐linked immunosorbent spot
IMAC: immobilized metal affinity chromatography
LB: Luria–Bertani medium
LNP: lipid nanoparticle
max: maximum
mRNA: messenger ribonucleic acid
MWCO: molecular weight cutoff
MW: molecular weight
PBS: phosphate‐buffered saline
PES: polyethersulfone
PNGase F: peptide N‐glycosidase F
RBD: receptor‐binding domain
RT: room temperature
SARS‐CoV‐2: severe acute respiratory syndrome coronavirus 2
SDS‐PAGE: sodium dodecyl sulfate‐polyacrylamide gel electrophoresis
SEC: size‐exclusion chromatography
SP: signal peptide
TC: tissue culture
ε: molecular extinction coefficient

The Severe Acute Respiratory Syndrome Coronavirus 2 (SARS‐CoV‐2 [1]) virus was responsible for the outbreak of the Coronavirus disease 2019 (COVID‐19) pandemic, which caused more than 770 million cases and almost 7 million deaths globally, to date (November 22, 2023 [2]). SARS‐CoV‐2 carries a single‐stranded positive‐sense RNA genome [3], which is encapsulated by an envelope consisting of a membrane and four structural proteins: the Spike (S), Envelope (E), Membrane (M), and Nucleocapsid (N) [4], forming the *corona* (crown) of the virion. Spike is the largest structural glycoprotein, functioning as a homotrimer complex on the virus' surface [5]. The Spike trimer is able to interact with the host cell receptor angiotensin‐converting enzyme 2 (ACE2), which is required for viral entry to the cell [4]. Therefore, Spike became the major target for research and vaccine development. Spike is synthesized as a single polypeptide, which is then split into the N‐terminal S1 and C‐terminal S2 subunits by the furin‐like protease in the Golgi apparatus [6]. Each Spike contains a C‐terminal transmembrane (TM) helix, which anchors the complex to the viral envelope (Fig. 1A). The largest, external part of the molecule, called the ectodomain, is required for trimer formation and virus internalization (Fig. 1A) [6]. The ectodomain comprises Spike's receptor‐binding domain (RBD), which directly engages the ACE2 receptor to mediate viral entry [7]. Moreover, RBD is the primary target of the multiple potent neutralizing antibodies that terminate the physical interaction between RBD and ACE2, and mediate immune response and virus neutralization [8, 9, 10]. Therefore, Spike's RBD (including its mutant forms) became a popular research and bio‐industrial target to study SARS‐CoV‐2. The RBD was used to establish biosensors to detect viral infection and seroconversion [11, 12] for research, preventive or diagnostic purposes, and to develop vaccines and therapies against viral infection and the COVID‐19 disease [10, 13, 14].

**Fig. 1:**
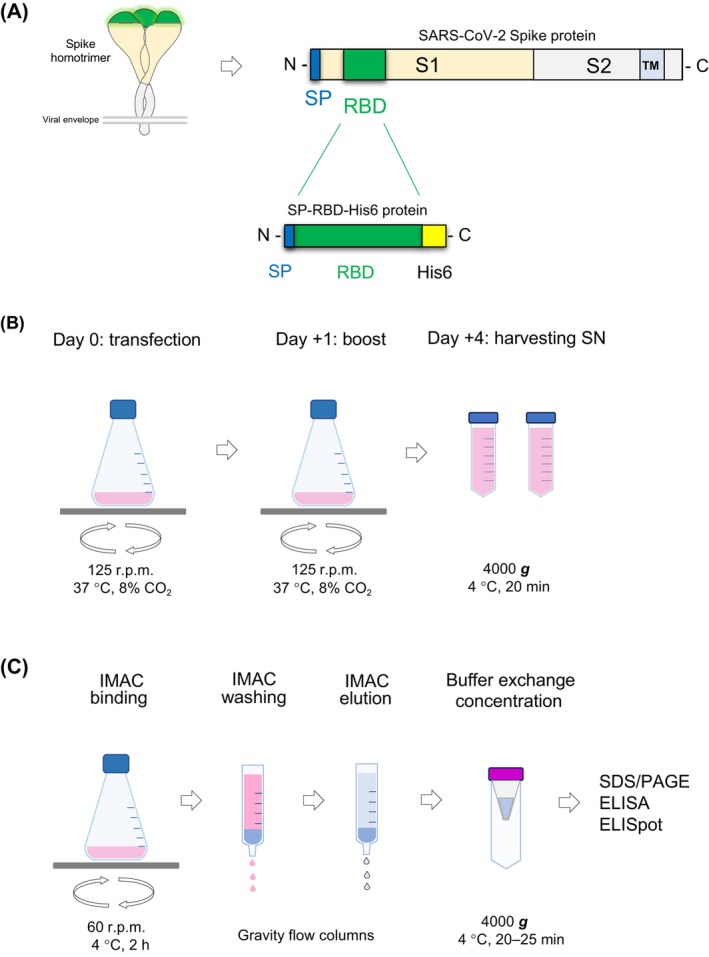
Expression and purification of recombinant receptor‐binding domain (RBD) from Expi293F mammalian cell culture supernatant. (A) Schematic representation of the homotrimer Spike complex anchored to the viral envelope (left). Domain and motif architecture of Spike is shown in the right panel: SP: signal peptide (dark blue); RBD: receptor‐binding domain (green); S1 subunit (gold); S2 subunit (gray); TM: transmembrane helix (light blue). The chimeric protein SP‐RBD^His6^ consists of the signal peptide fused to the RBD domain of Spike and a C‐terminal hexa histidine‐tag (His6, yellow). Steps of RBD expression in transiently transfected Expi293F cells (B) and IMAC purification (C) from cell culture supernatant. ELISA, enzyme‐linked immunosorbent assay; ELISpot, enzyme‐linked immunosorbent spot; IMAC, immobilized metal affinity chromatography.

The production of recombinant RBD or its variants is cost‐efficient, and the purified product is suitable for both *in vitro* and *in vivo* use. Over the years, several heterologous expression systems have been developed to produce recombinant RBD. It can be expressed in bacteria, purified from inclusion bodies, and refolded [15, 16, 17, 18]. However, bacterially produced RBD has some usage limits due to the lack of functionally relevant glycosylation, or heterotrimeric complex formation. The expression of RBD in lower eukaryotes partially solves this problem. A high yield of RBD can be achieved in baculovirus‐mediated [19] or transiently transfected [20] insect cells, or in yeast systems [21]. Although structurally functional, these recombinant RBDs are differently glycosylated than that of the viral RBD in humans, making them less advantageous in functional assays. The production of RBD in mammalian cell cultures, including Chinese hamster ovary (CHO) or human embryonic kidney (HEK293) cells, has overcome this problem. The receptor‐binding domain is properly glycosylated in mammalian cells, easy to purify from the cell culture supernatant, reaches high quantities, and is capable of homodimer and homotrimer formation [11, 22, 23, 24]; hence, it is suitable for serological assays as well as *in vivo* experiments or diagnostic purposes.

In this research protocol, we provide a detailed, step‐by‐step guide for the production and one‐step purification (Fig. 1) of homomeric and glycosylated (Fig. 2), as well as structurally intact (Fig. 3, Table 1), RBD in transiently transfected Expi293F human cells, which is suitable for immunological assay (Fig. 4). Up to 80 mg of RBD can be produced in 1 L of cell culture in only 4 days. The recombinant RBD purified from the clarified cell culture supernatant by immobilized metal affinity chromatography (IMAC) is extremely pure (> 98% based on SDS/PAGE analysis, Fig. 2); therefore, it does not require additional clarification steps. Moreover, it forms homodimers and homotrimers (Fig. 2); therefore, we could successfully use it in immunological experiments, such as the enzyme‐linked immunosorbent assay (ELISA, Fig. 4). This protocol can also be used to produce different variants of RBD.

**Fig. 2:**
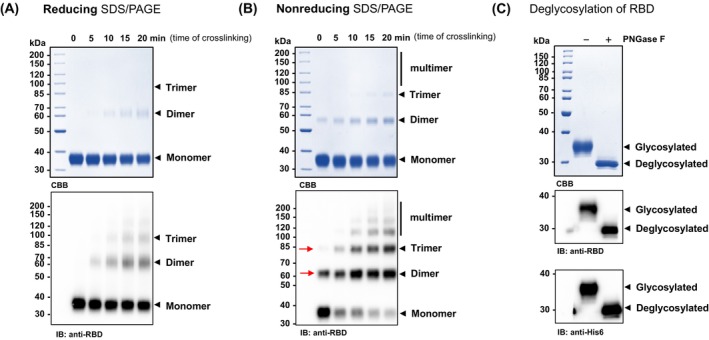
Recombinant receptor‐binding domain (RBD) is glycosylated and forms homomeric complexes. Purified RBD was treated with a disuccinimidyl suberate (DSS) crosslinker and incubated at room temperature for the indicated time points (0–20 min). The reaction was quenched and samples from each time point were subjected to reducing (A) or nonreducing (B) SDS/PAGE analysis followed by Coomassie Brilliant Blue (CBB) staining (upper panels), or immunoblotting (IB, lower panels) using an anti‐RBD monoclonal antibody (generated in the house). Zero (0) minutes served as the negative control (non‐DSS‐treated). Under nonreducing conditions non‐crosslinked RBD forms homodimers and homotrimers (Panel B, 0 min, red arrows). Under reducing and nonreducing conditions, crosslinked RBD forms homodimer, homotrimer, and even multimeric high‐molecular‐weight complexes in a time‐dependent manner. This suggests that the dimeric/trimeric forms of RBD are present in the native, purified samples. Besides noncovalent intermolecular interactions, the complexes are stabilized by disulfide bonds, as well. (C) The N‐glycosylation of RBD was tested by PNGase F‐treatment. Samples were incubated in the absence (−) or presence (+) of PNGase F enzyme under denaturing conditions for 30 min at room temperature and subjected to SDS/PAGE analysis followed by Coomassie Brilliant Blue staining (CBB, upper panel) and immunoblotting (IB, lower panels) with anti‐RBD and anti‐His6 antibodies, respectively. This shows that the electrophoretic mobility of the PNGase F‐treated RBD protein changes significantly in the gel due to the removal of N‐glycans, suggesting that the purified RBD protein is N‐glycosylated.

**Fig. 3:**
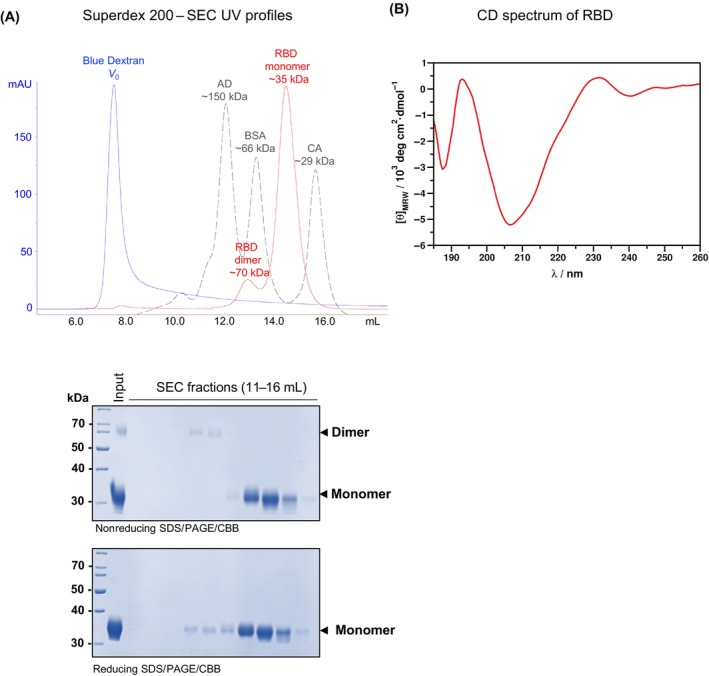
Purified receptor‐binding domain (RBD) is structurally intact. (A) Purified RBD was subjected to Superdex 200 (HR 10/30) size‐exclusion chromatography (SEC) column precalibrated with SEC protein standards (gray dashed line: alcohol dehydrogenase (AD, ~150 kDa), bovine serum albumin (BSA; ~66 kDa), and carbonic anhydrase (CA, ~29 kDa)). The void volume (*V*_0_ = ~7 mL) of the column was determined by Blue Dextran 2000 (blue line). Every other SEC fraction (between ~11 and 16 mL, red line) was analyzed by nonreducing (upper panel) and reducing (lower panel) SDS/PAGE followed by Coomassie Brilliant Blue staining (CBB) revealing that both dimeric and monomeric RBD (the two major forms under native conditions, Fig. 2B) run around their expected sizes (~70 and ~ 35 kDa, respectively, red line and gels) and do not form soluble aggregates. (B) The secondary structure of the purified RBD protein was examined by CD spectroscopy. The CD spectrum is in good agreement with the spectrum published previously for the same protein expressed in mammalian cells [30]. The specific contributions emerging from the constituent secondary structural elements determined by spectral deconvolution is presented in Table 1 and are also in good agreement with those calculated from the high‐resolution coordinates of the protein of subject [29].

**Table 1:** Fractions of secondary structural contributions expressed as percentages, as determined by CD spectroscopy and calculated from the high‐resolution structure of SARS‐CoV2 spike protein RBD domain [29].

	Helix (α and 3_10_)	β‐Strand	Turns and bends	Unordered
CD‐derived structure	12.0	36.0	25.0	27.0
Cryo‐EM structure (7DZW)	11.7	26.5	24.7	37.1

**Fig. 4:**
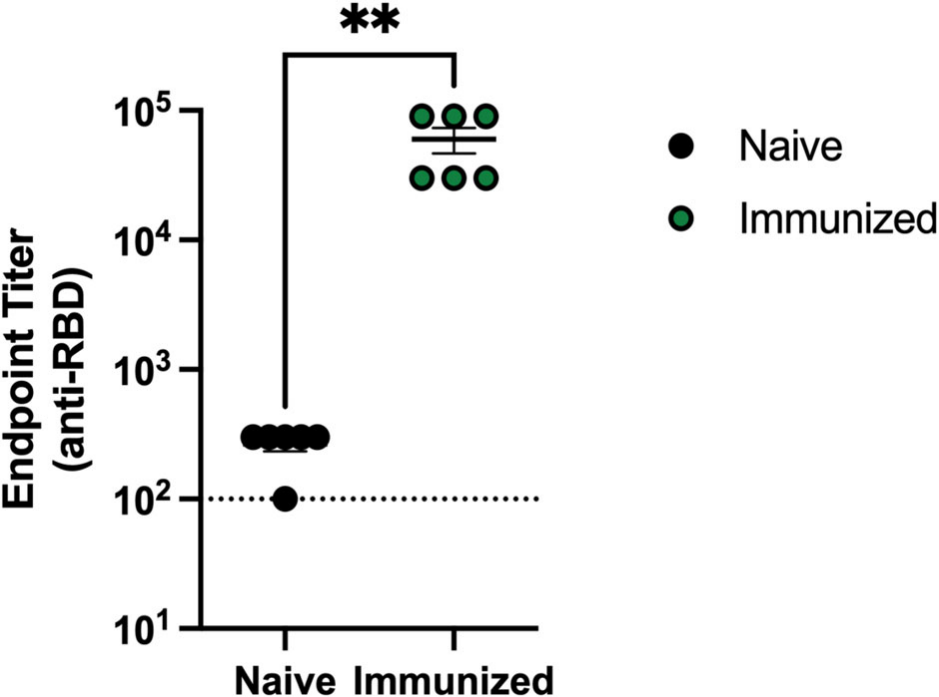
Receptor‐binding domain (RBD)‐specific IgG titers in mouse sera were determined after immunization with a spike‐encoding nucleoside‐modified mRNA‐LNP vaccine. Mouse immunizations and ELISA were performed and nucleoside‐modified mRNA‐LNP vaccines were generated as described in our previous studies [31, 32]. Eight‐week‐old female BALB/c mice were immunized intramuscularly with 3 μg of full‐length spike‐encoding mRNA encapsulated with lipid nanoparticle (LNP). Four weeks postimmunization, sera were collected, and RBD‐specific immunoglobulin (IgG) titers were determined by endpoint dilution ELISA. *n* = 6 mice. Error bars are SEM. Each symbol represents one animal. The limit of detection is shown as a horizontal dotted line on the graph. Statistical analysis: unpaired *t*‐test; **P‐value ≤ 0.01.

## Materials

### Endotoxin‐free plasmid DNA preparation

1. pCDNA3.1[SP‐RBD^His6^] plasmid:
  DNA sequence encoding the signal peptide (SP, aa 1–14) and the RBD (aa 319–541) of SARS‐CoV‐2‐Spike protein (NCBI accession no.: YP_009724390) in fusion with a C‐terminal hexa histidine‐tag (His6) and a stop codon (Fig. 1A and Tables 2 and 3) was optimized to mammalian codon preference, produced by gene synthesis (GenScript, Piscataway, NJ, USA), and subcloned into the pCDNA3.1(−) mammalian expression plasmid (Invitrogen, Waltham, MA, USA #V79520).
2. Subcloning efficiency chemically competent DH5α *Escherichia coli* cells (Invitrogen #18265017).
3. Luria–Bertani medium (LB broth): 10 g tryptone, 5 g NaCl, 5 g yeast extract in 1 L ddH_2_O, and pH 7.2–7.5 (autoclave for 15 min at 120 °C).
4. Luria–Bertani broth with 1.5% agar–agar plates, supplemented with 100 μg·mL^−1^ carbenicillin (Serva, Heidelberg, Germany #15875.03).
5. 1‐L borosilicate glass Erlenmeyer flasks and centrifuge tubes.
6. Phosphate‐buffered saline (PBS): 137 mm NaCl, 2.6 mm KCl, 8.7 mm Na_2_HPO_4_, 1.7 mm NaH_2_PO_4_. Filter‐sterilize and store at 4 °C.
7. Laboratory shaker.
8. Refrigerated centrifuge.
9. Endotoxin‐Free Maxi Plasmid Purification Kit (Thermo Scientific, Waltham, MA, USA #A33073).
10. Spectrophotometer.

**Table 2:** Amino acid sequence of the SP‐RBD^His6^ protein. The signal peptide (SP) and the hexa histidine‐tag (His6) are underlined.

MFVFLVLLPLVSSQRVQPTESIVRFPNITNLCPFGEVFNATRFASVYAWNRKRISNCVADYSVLYNSASFSTFKCYGVSPTKLNDLCFTNVYADSFVIRGDEVRQIAPGQTGKIADYNYKLPDDFTGCVIAWNSNNLDSKVGGNYNYLYRLFRKSNLKPFERDISTEIYQAGSTPCNGVEGFNCYFPLQSYGFQPTNGVGYQPYRVVVLSFELLHAPATVCGPKKSTNLVKNKCVNFGHHHHHH

**Table 3:** Nucleotide sequence of the SP‐RBD^His6^ protein‐encoding synthetic DNA. The signal peptide (SP) and the hexa histidine‐tag (His6) are underlined.

ATGTTCGTGTTCCTGGTGCTGCTGCCCCTGGTGTCCTCCCAGCGCGTGCAGCCCACCGAGTCCATCGTGCGCTTCCCCAACATCACCAACCTGTGCCCCTTCGGCGAGGTGTTCAACGCCACCCGCTTCGCCTCCGTGTACGCCTGGAACCGCAAGCGCATCTCCAACTGCGTGGCCGACTACTCCGTGCTGTACAACTCCGCCTCCTTCTCCACCTTCAAGTGCTACGGCGTGTCCCCCACCAAGCTGAACGACCTGTGCTTCACCAACGTGTACGCCGACTCCTTCGTGATCCGCGGCGACGAGGTGCGCCAGATCGCCCCCGGCCAGACCGGCAAGATCGCCGACTACAACTACAAGCTGCCCGACGACTTCACCGGCTGCGTGATCGCCTGGAACTCCAACAACCTGGACTCCAAGGTGGGCGGCAACTACAACTACCTGTACCGCCTGTTCCGCAAGTCCAACCTGAAGCCCTTCGAGCGCGACATCTCCACCGAGATCTACCAGGCCGGCTCCACCCCCTGCAACGGCGTGGAGGGCTTCAACTGCTACTTCCCCCTGCAGTCCTACGGCTTCCAGCCCACCAACGGCGTGGGCTACCAGCCCTACCGCGTGGTGGTGCTGTCCTTCGAGCTGCTGCACGCCCCCGCCACCGTGTGCGGCCCCAAGAAGTCCACCAACCTGGTGAAGAACAAGTGCGTGAACTTCGGCCATCATCACCATCACCATTGA

### Protein expression in Expi293F mammalian cells

1. 1 vial (10^7^) of cryopreserved Expi293F mammalian cells (Gibco, Waltham, MA, USA #A14527).
2. Expi293 Expression Medium (Gibco #A1435101).
3. ExpiFectamine 293 transfection kit (Gibco #A14524).
4. Opti‐MEM I Reduced Serum Medium (Gibco #11058021).
5. Cell counter (automated or manual).
6. Humidified CO_2_ incubator with thermoregulation.
7. CO_2_ resistant orbital (1.9 cm/0.75 in) shaker with flask clamps or self‐adhesive platform (Thermo Scientific #88881102).
8. Regular tissue culture (TC) hood (e.g., Class II Biological Safety Cabinet) with UV lamp.
9. 37 °C water bath.
10. 125‐mL and 1‐L single‐use PETG tissue culture Erlenmeyer flasks with plain bottom and vented closure (Nalgene, Waltham, MA, USA #4115‐0125/1000) (Tips & Tricks 1).
11. Sterile serological pipettes with cotton plug, and pipette aid.
12. Sterile and filtered pipette tips, and single‐channel pipettes.
13. Sterile microfuge tubes, 15‐ and 50‐mL conical tubes.

### Protein purification, buffer exchange, and concentration

1. Syringe (cellulose acetate membrane, 0.22 μm) and 250–1000 mL vacuum (PES, polyethersulfone membrane, 0.22 μm) filter units for sterile filtration.
2. 2‐ and 5‐mL Luer‐lock syringes.
3. Vacuum device or aspirator.
4. RBD‐W buffer: 50 mm sodium phosphate pH 8.0, 300 mm NaCl, 20 mm imidazole (Tips & Tricks 2). Filter‐sterilize and store at 4 °C for 1 year.
5. RBD‐E buffer: 50 mm sodium phosphate pH 8.0, 300 mm NaCl, 300 mm imidazole. Filter‐sterilize and store at 4 °C for 1 year.
6. Ni Sepharose 6 Fast Flow resin for IMAC purification (Cytiva, Washington, WA, USA #17531802) (Tips & Tricks 3).
7. Polypropylene empty PD‐10 (Cytiva #17043501) or similar chromatographic columns.
8. Amicon Ultra‐15 Centrifugal Filter Unit with 10 kDa MWCO (Millipore, Burlington, MA, USA #UFC9010)
9. Liquid nitrogen.

## Methods

### Preparation of endotoxin‐free plasmid DNA

1. Transform chemically competent *E. coli* cells with 50 ng pCDNA3.1‐SP‐RBD^His6^ plasmid, incubate on ice for 20 min, spread the bacteria onto an LB agar plate, and incubate at 37 °C for 14–16 h.
2. Inoculate 200 mL of LB broth (in 1‐L glass flask) supplemented with 100 μg·mL^−1^ carbenicillin with few colonies of transformed bacteria and grow at 37 °C for 14–16 h with 280 r.p.m.
3. Purify plasmid DNA following the endotoxin‐free maxi prep's guidelines.
4. Filter‐sterilize the plasmid DNA and store it in small aliquots (200 μg each) at −20 °C.

### Thawing, culturing, and expansion of Expi293F cells

1. Thawing: thaw one vial (10^7^) of low passage number (< 20) cryopreserved Expi293F cells in a 37 °C water bath (Tips & Tricks 4).
2. Transfer the cells into 30 mL prewarmed Expi293 Expression medium in a 125‐mL flask.
3. Incubate the cells in a 37 °C tissue culture incubator (> 80% relative humidity) with 8% CO_2_ on a shaker at 125 r.p.m. for 4–5 days (Tips & Tricks 5).
4. Count cells and calculate cell viability using automated or manual cell counters. Perform the first subculture when cell density reaches 2–3 × 10^6^ viable cells·mL^−1^. Cell viability should be > 90%.
5. First subculturing: transfer 400 000 viable cells to a prewarmed medium (30 mL of final volume) in a new 125‐mL flask and incubate the culture as above (step 3) for 2–3 days.
6. Count cells and perform the second subculture when cell density reaches 2–3 × 10^6^ viable cells·mL^−1^. Cell viability should be > 90%.
7. Second subculturing: transfer 400 000 viable cells to a prewarmed medium (30 mL of final volume) in a new 125‐mL flask and incubate the culture as above (step 3) for 2–3 days.
8. Count cells and perform the third subculture (expansion) when cell density reaches 3–5 × 10^6^ viable cells·mL^−1^. Cell viability should be > 95% (Tips & Tricks 6).
9. Expanding: transfer 500 000 viable cells to a prewarmed medium (150 mL of final volume) in a new 1‐L PETG tissue culture flask and incubate the culture as above (step 3) for 2–3 days until cell density reaches approximately 3–5 × 10^6^ viable cells·mL^−1^ (Tips & Tricks 7).

### Transient transfection of Expi293F cells

1. Seeding cells (Day −1): One day before transfection determine cell count and viability, then seed 495 million (4.95 × 10^8^) viable cells in 180 mL of prewarmed medium in a clean 1‐L PETG flask (final cell density and viability should be 2.75 × 10^6^ viable cells·mL^−1^ and > 95%, respectively) and grow them overnight (12–16 h) as above (Tips & Tricks 8).
2. Transfection (Day 0):
  2.1. Determine cell count and viability. It should be 4–6 × 10^6^ viable cells·mL^−1^ and > 95%, respectively. Gently dilute cells to a final density of 3 × 10^6^ viable cells·mL^−1^ in 200 mL of prewarmed medium (600 million cells) in a clean 1‐L PETG flask and allow the cells to adapt to the medium in a 37 °C tissue culture incubator (> 80% relative humidity) with 8% CO_2_ on a shaker at 125 r.p.m. for 15–30 min.
  2.2. Mix the ExpiFectamine 293 transfection reagent by gentle inversion several times, before adding 640 μL into 11.2 mL room temperature (RT) Opti‐MEM I reduced serum medium in a 50‐mL conical tube (Tube 1). Mix by gentle swirling and incubate at RT for 5 min.
  2.3. Dilute 200 μg plasmid DNA (pCDNA3.1‐SP‐RBD^His6^) in 12 mL room temperature Opti‐MEM I medium in a 50‐mL conical tube (Tube 2) and mix several times by gently swirling. Incubate at RT for 5 min.
  2.4. Using a sterile serological pipette add Tube 1 to Tube 2, mix by gentle swirling, and incubate at RT for 10 min.
  2.5. Slowly, drop‐by‐drop transfer the DNA/transfection complex mixture to the cells. Gently shake the culture during complex addition.
  2.6. Grow the transfected cells in a 37 °C tissue culture incubator (> 80% relative humidity) with 8% CO_2_ on a shaker at 125 r.p.m. for 18–22 h.
3. Boosting protein expression (Day +1):
  3.1. Mix 1.2 mL ExpiFectamine 293 Enhancer 1 and 12 mL ExpiFectamine 293 Enhancer 2 in a 50‐mL conical tube by gentle inversion.
  3.2. Slowly, drop‐by‐drop transfer the enhancer mixture to the cells (grown 18–22 h post‐transfection [step 2.6]). Gently shake the culture during booster addition.
  3.3. Immediately return the flask to the 37 °C tissue culture incubator (> 80% relative humidity) with 8% CO_2_ and grow the cells on a shaker at 125 r.p.m. for 3 days (Tips &Tricks 9).
4. Harvesting secreted RBD (Day +4):
  4.1. 96‐h post‐transfection harvest the cells by centrifugation at 4000 ***g***, 4 °C, for 20 min, in a swinging‐bucket rotor.
  4.2. Carefully pour the cell culture supernatant (~220–230 mL, this contains the secreted RBD^His6^) into a sterile 1‐L glass flask without disturbing the pellet. Discard the cell pellet.
  4.3. Slowly add 14 mL of RBD‐E buffer into the clarified cell culture supernatant and mix evenly by gently shaking the flask (Tips &Tricks 10).
  4.4. With a vacuum filter unit, filter the mixture into a sterile 1‐L glass flask to remove residual cells and aggregates.

### IMAC purification of RBD^His6^

1. Thoroughly mix the Ni Sepharose 6 Fast Flow resin (Tips &Tricks 3), take out 6 mL slurry (50% bed volume), and mix with 40 mL RBD‐W buffer in a 50‐mL conical tube to equilibrate the resin.
2. Centrifuge the tube at 800 ***g***, 4 °C, for 5 min in a swinging‐bucket rotor with low deceleration (e.g., set the brake to 7 out of 10) to avoid mixing the resin beads due to turbulence.
3. Carefully pour off and discard the buffer and resuspend the matrix in 10 mL of RBD‐W buffer.
4. Transfer the IMAC resin into the 1‐L glass flask containing the cell culture supernatant from Step 4.4.
5. Incubate the flask on an orbital shaker at 60 r.p.m., for 2 h, at 4 °C.
6. Fix an empty PD‐10 (or similar) chromatographic column to a stand.
7. Continuously transfer the resin/medium suspension from the flask to the column and let the medium flow through the resin. Discard the flow‐through (Tips & Tricks 11).
8. Step‐wash the resin in the column with 5 × 10 mL RBD‐W buffer. Let the buffer flow through the resin. Discard the washing buffer.
9. Step‐elute RBD^His6^ with 5 × 3 mL RBD‐E buffer into a sterile prechilled 15‐mL conical tube.

### Buffer exchange, concentration, and long‐term storage of RBD^His6^

1. Add 5 mL of sterile PBS into a 15‐mL centrifugal filter device (concentrator) with 10 kDa MWCO and centrifuge at 4000 ***g***, 4 °C, for 5 min in a swinging‐bucket rotor. Discard the flow‐through from the filtrate collection tube.
2. Transfer the eluted RBD^His6^ (15 mL) from Step 9 into the filter device and centrifuge at 4000 ***g***, 4 °C, for 15–25 min until the sample in the device is concentrated to circa 5 mL. Discard the flow‐through from the filtrate collection tube.
3. Add 10 mL of sterile ice‐cold PBS to the concentrated sample, close the lid, mix by gentle inversion several times, and centrifuge at 4000 ***g***, 4 °C, for 15–25 min until the sample is concentrated to 4–5 mL. Discard the flow‐through from the filtrate collection tube.
4. Repeat Step 3 five times (Tips & Tricks 12).
5. Collect the buffer exchanged and concentrated RBD^His6^ sample from the device (circa 5 mL) and transfer it to a prechilled tube.
6. Centrifuge (12 000 ***g***, 4 °C, 10 min) the sample to remove any precipitates formed during the buffer exchange and concentration. Save and transfer the supernatant to a new prechilled tube.
7. Measure the concentration of the purified RBD^His6^ with a spectrophotometer at 280 nm (Tips & Tricks 13):
  7.1. Set the MW to 25.98 kDa.
  7.2. Set the ε/1000 to 33.850 m ^−1^·cm^−1^.
  7.3. Set the baseline correction, if available, to 340 nm.
  7.4. Use PBS as a blank.
  7.5. Measure the concentration of the purified sample from Step 6.
8. Slowly adjust the concentration of RBD^His6^ to 1 mg·mL^−1^ with sterile and cold PBS (Tips & Tricks 14), mix gently, and test the integrity of the protein by SDS/PAGE (Fig. 2) [25].
9. Filter‐sterilize the purified proteins (optional).
10. Prepare aliquots as desired (into prechilled microfuge tubes).
11. Flash‐freeze the aliquots in liquid nitrogen and store the samples at −80 °C.

### Tips & Tricks

1. Sterile borosilicate glass Erlenmeyer flasks can also be used with sterile metal caps (VWR, Radnor, PA, USA #391‐0951). Never use flasks that have been in contact with bacteria, because of endotoxin hazards. If reused, wash flasks and caps extensively, rinse in ultrapure water, and dry sterilize at 180 °C for 4–6 h.
2. All chemicals should be at high purity (e.g., molecular biology or cell culture grade).
3. Other types of high‐capacity IMAC resins can also be used, including Ni‐NTA, cobalt resin, or TALON matrix from different vendors.
4. Basic mammalian cell culture maintenance techniques [26] and the guidelines of the Expi293 Expression System manual (https://www.thermofisher.com) should be followed. All steps must be carried out carefully and precisely under sterile conditions. Do not use antibiotics (e.g., penicillin or streptomycin) or other additives (e.g., l‐glutamine and surfactants) in the cell culture medium.
5. If a 25‐ or 50‐mm‐diameter orbital shaker is used, shake the cells at 120 or 95 r.p.m., respectively.
6. If cell viability is lower than 95% or cell growth is slow, repeat Steps 6–7 and perform 1 or 2 extra subculturing steps in 30 mL medium.
7. It is recommended to continue subculturing in a 30 mL volume (in 125‐mL flasks), too, in parallel to the expanded cells to be used for transfection. This parallel culture will serve as a reserve material for another transfection or can be used for cryopreservation of Expi293F cells (follow the guidelines of the Expi293F cells' manual).
8. If smaller (e.g., for pilot experiments) or larger (e.g., for high‐scale protein production) volumes are needed, use smaller or larger flasks, and perform the transfection steps according to the Expi293 Expression System manual.
9. During the 4‐day protein expression and secretion, Expi293F cells can reach extremely high densities; therefore, the culture may turn whitish and form cell clumps and rims over the medium. These are normal phenomena and do not affect protein production.
10. The addition of RBD‐E buffer is necessary to adjust the pH of the cell culture media as well as to set the imidazole concentration to ~20 mm to decrease nonspecific binding during the IMAC purification of RBD. The color of the medium should be pinkish.
11. It is recommended to perform the purification at 4 °C. If this is not possible, place the suspension and buffers on ice and collect the eluted samples into prechilled tubes placed on ice. The resin/medium suspension is transferred from the flask to the columns by a serological pipette or a peristaltic pump.
12. Other types of buffer exchange and/or concentration can also be followed, including tangential flow filtration, dialysis, or size‐exclusion chromatography [23].
13. The concentration of the intact and purified RBD^His6^ can be measured precisely with a spectrophotometer. We use a NanoDrop OneC, however, any type of spectrophotometer that measures at 280 nm can be used. Based on the primary sequence of the purified protein (excluding the signal peptide), the Mw and ε of any RBD variant can be easily calculated with the ProtParam online tool (https://web.expasy.org/protparam/) [27]. If a spectrophotometer is not available, other types of protein concentration measurement methods may also be used [28].
14. We keep the concentration of purified RBD between 0.5 and 2 mg·mL^−1^ and try to avoid several cycles of freezing and thawing of the samples. When needed, we thaw the frozen stocks on ice and centrifuge (12 000 ***g***, 4 °C, 10 min) before use in any assay.

## Conflict of interest

NP is named on patents describing the use of nucleoside‐modified mRNA in lipid nanoparticles as a vaccine platform. He has disclosed those interests fully to the University of Pennsylvania, and he has in place an approved plan for managing any potential conflicts arising from licensing of those patents. NP served on the mRNA strategic advisory board of Sanofi Pasteur in 2022. NP is a member of the Scientific Advisory Board of AldexChem and Bionet‐Asia.

## Author contributions

EA and ZL performed cloning, protein expression and purification. CB, AM, TM, and NP generated mRNA‐LNP vaccines, immunized mice, and performed ELISA assay. ZL, AB, TM, and NP created the figures. NP and ZL wrote the research protocol and contributed to the funding. AB performed CD spectroscopy and contributed to the funding.

## Data Availability

Datasets presented in the figures and the experimental setups are available upon request. pCDNA3.1[SP‐RBD^His6^] plasmid is available upon request for nonprofit research organizations.
